# Improvement of the Fluorescence Intensity during a Flow Cytometric Analysis for Rice Protoplasts by Localization of a Green Fluorescent Protein into Chloroplasts

**DOI:** 10.3390/ijms16010788

**Published:** 2014-12-31

**Authors:** Min Kyoung You, Sun-Hyung Lim, Min-Jin Kim, Ye Sol Jeong, Mi-Gi Lee, Sun-Hwa Ha

**Affiliations:** 1 National Academy of Agricultural Science, Rural Development Administration, Jeonju 560-500, Korea; E-Mails: minkyou@khu.ac.kr (M.K.Y.); limsh2@korea.kr (S.H.L.); sweetwindy@ajou.ac.kr (M.J.K.); yesol@korea.ac.kr (Y.S.J.); 2 Graduate School of Biotechnology and Crop Biotech Institute, Kyung Hee University, Yongin 446-701, Korea; 3 Gyeonggi Bio-Center, Gyeonggi Institute of Science & Technology Promotion, Suwon 443-270, Korea; E-Mail: migi@gstep.re.kr

**Keywords:** chloroplast, flow cytometric analysis (FCA), protoplast, rice, transit peptide, fluorescence-activated cell sorting (FACS), synthetic green fluorescent protein (sGFP)

## Abstract

Protoplasts have been a useful unicellular system for various molecular biological analyses based on transient expression and single cell analysis using fluorescence-activated cell sorting (FACS), widely used as a powerful method in functional genomics. Despite the versatility of these methods, some limits based on low fluorescence intensity of a flow cytometric analysis (FCA) using protoplasts have been reported. In this study, the chloroplast targeting of fluorescent proteins (FPs) led to an eight-fold increase in fluorescence intensity and a 4.5-fold increase of transfection ratio from 14.7% to 65.7% as compared with their targeting into the cytoplasm. Moreover, the plot data of FCA shows that 83.3% of the K-sGFP population is under the threshold level, regarded as a non-transgenic population with background signals, while 65.7% of the K-sGFP population is spread on overall intervals. To investigate the reason underlying this finding, mRNA/protein levels and transfection efficiency were analyzed, and results suggest that mRNA/protein levels and transfection ratio are not much different between K-sGFP and KR-sGFP. From those results, we hypothesized that the difference of fluorescence intensity is not only derived from cellular events such as molecular level or transfection efficiency. Taken together, we suggest that the translocation of FPs into chloroplasts contributes to the improvement of fluorescence intensity in FCA and, apparently, plays an important role in minimizing the loss of the transfected population. Our study could be usefully applicable for highly sensitive FACS and FCA-investigations of green tissue.

## 1. Introduction

A protoplast is a naked plant cell bounded only by the cell membrane that is obtained by enzymatic treatment to remove the cell wall. The structural property of protoplasts facilitates the study of transient expression systems. Recently, protoplasts have been reported to retain their tissue- and cell-specific features within the time frame of a transient expression assay [1,2]. This property is combined with the advantage of a fluorescence-activated cell sorting (FACS) that sorts protoplasts only having a fluorescent signal [2,3] to facilitate molecular biological studies on the expression profiling of local tissues, such as the *Arabidopsis* root quiescent center and sperm cells [4,5]. Those studies have highlighted the potential of single cell-based FACS using protoplasts. Also, single cell-based flow cytometric analysis (FCA) has been successfully applied to *in vivo* analysis of protein-protein interactions [6,7], and promoter activity [8,9], *etc*.

Hagenbeek and Rock (2001) [9] reported the usefulness of FCA for the promoter analysis system using protoplasts, and described the advantages and disadvantages of FCA, in detail. As a major disadvantage, they showed that the fluorescence intensity of GFP was unusually detected to be as low as the threshold level of background signals, and subsequently, it caused the transfection ratio determined to be as low as 4%, which represented the population size of protoplasts expressing GFP-fluorescent signals on FCA. Since the average transfection efficiency determined on hemocytometer analysis has been reported to be *ca.* 40%–60% [9], a large number of transfected protoplasts might supposedly have been missed on FCA. It could be a bottleneck on application of plant protoplast system to various FCA or FACS. To overcome these limitations of FCA using protoplasts, higher DNA quantities (10–130 μg) were used, but the transfection ratio, (amounting to 6%), was not markedly improved. Thus, the low fluorescence intensity of FCA using plant protoplasts remains a challenging limitation.

As chloroplasts are an isolated space bounded by an envelope membrane and a single plant cell comprises many (tens to hundreds) of chloroplasts [10], they have been reported to be an excellent reservoir for producing various recombinant proteins [11]. To localize the fluorescent protein (FP) in the chloroplast, the transit peptide originated from the small subunit of the enzyme ribulose-1,5-bisphosphate carboxylase/oxygenase (RuBisCo) of rice [12], used to localize various recombinant proteins into chloroplasts, was selected. Chloroplasts have been reported to have a limited set of protein degradation pathways in the stroma compared with that of the cytoplasm; it has been an advantage of chloroplasts that recombinant proteins localized within them could be kept more stable than those expressed in the cytoplasm [13,14].

Additionally, a Kozak (Kz) sequence, a defined consensus sequence adjacent to the ATG initiation codon in eukaryotic mRNAs, was considered for the improvement of the translation efficiency, which has been reported to mediate efficient initiation of translation by ribosomes [15]. The Kz sequences have been identified in various eukaryotes including vertebrates, yeasts, protozoa, and plants [16,17,18].

Based on the above advantages, the chloroplast and Kz sequence in this study were adopted for the localization space of FPs and the expression efficiency, respectively. The useful strategy of FCA using rice protoplasts was developed for increasing the fluorescence intensity of FPs analyzed by flow cytometry.

## 2. Results

### 2.1. Expression of a Green Fluorescent Protein in Rice Protoplasts

**Figure 1:**
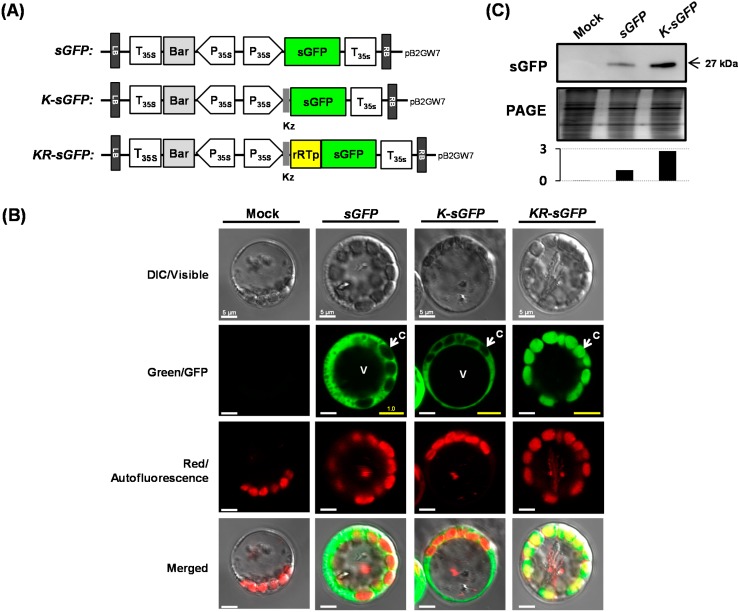
Subcellular localization and expression efficiency of sGFPs in rice protoplasts. (**A**) Three kinds of sGFP chimera were cloned into a pB2GW7 vector to construct *sGFP*, *K-sGFP*, and *KR-sGFP* for use in PEG-mediated transfection into rice protoplasts. Kz: Kozak sequence; rRTp: a transit peptide of rice RuBisCo small subunit; (**B**) Individual and merged images of GFP, chlorophyll autofluorescence, and visible protoplasts. C: chloroplast; V: vacuole. The images were acquired at 630× magnification using a confocal microscope. The yellow signals are the merged image of the green fluorescent signals from the sGFP proteins; the red fluorescent signals from chlorophyll in the chloroplasts. The PEG-transformed protoplasts with no plasmid were used as a mock sample. The yellow scale bars on the images show the mean intensity ratio of the green fluorescence signals (for 10^5^ pixels), analyzed by a Histogram tool, and the white scale bars mean 5 μm and (**C**) The expression efficiency of proteins was compared using western blot analysis. Polyclonal rabbit antibody against sGFP was used at a titer of 1:5000 and the image of PAGE gel was used to show relative quantities of the loaded proteins. The graph shows the relative band intensities that were quantified using LAS4000 software.

A synthetic GFP (sGFP), a derivative with an increased brightness adjusted to plant systems [19], was used for FCA using rice protoplasts. Three kinds of sGFP-expressing vectors were constructed (see Figure 1A): *sGFP*, as a control construct; *K-sGFP*, a construct with plant-type Kz sequence near a translation start codon; and *KR-sGFP*, a construct with a Kz sequence and a transit peptide sequence of the rice RuBisCo small subunit (rRTp, R) for N-terminal fusion to sGFP. For the transfection with each vector DNAs, rice protoplasts were isolated from the 10-day-old rice seedlings and their fluorescence was examined under a confocal microscope. The results based on the green fluorescence showed that the sGFP signals from *sGFP* and *K-sGFP* were all dispersed into the cytoplasm and those from *KR-sGFP* were clearly localized into chloroplasts, supported by the exact match to red autofluorescence from chlorophylls (see Figure 1B). The mean fluorescence intensity of the images was analyzed by using a Histogram tool of the image acquisition software ZEN 2009 Light Edition, and the relative ratios of the green fluorescence intensities were introduced by the yellow scale bars on the images of Green/GFP in Figure 1B, which were normalized to it of the *sGFP*-transformed protoplasts. The confocal microscopic analysis showed that the localization of sGFP into chloroplasts did not have any effects on the fluorescence intensity of sGFP.

The effect of Kz sequence on sGFP expression level was also analyzed (see Figure 1C). Western blot analysis using a GFP antibody showed that the level of expression of *K-sGFP* proteins was a factor of 2.8 higher than that of *sGFP* proteins.

### 2.2. Differences on FCA between Cytoplasm- and Chloroplast Targeting of sGFP

**Figure 2:**
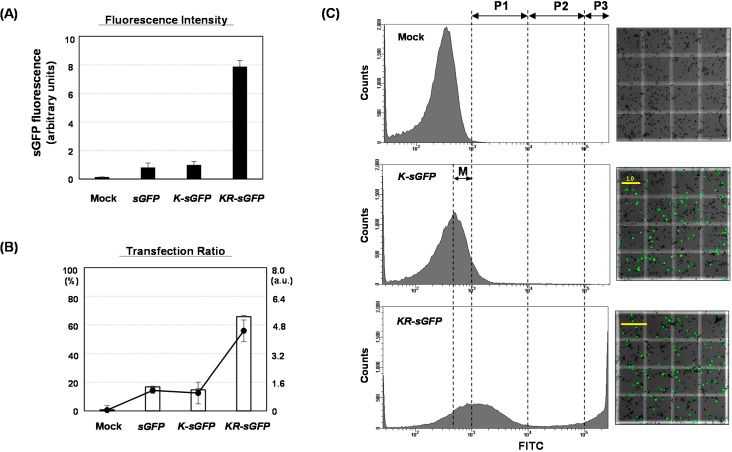
Comparative analysis of FCA using rice protoplasts among *sGFP*, *K-sGFP* and *KR-sGFP* constructs. Rice protoplasts were transfected with 5 μg plasmids of *sGFP*, *K-sGFP*, and *KR-sGFP* constructs. The FCAs were performed using single live cells gated by the side/forward scattering. (**A**) The fluorescence intensities were analyzed using the fluorescein isothiocyanate (FITC-A) channel of the flow cytometer and normalized by the average fluorescence intensity of the *K-sGFP* construct. The results are presented in arbitrary units; (**B**) The ratio of protoplasts with a fluorescence intensity exceeding the threshold value (10^3^) to the gated protoplasts was calculated on FCA. The left axis shows the percentage of the transfected protoplasts expressing sGFP and the right axis shows their arbitrary results. The error bars show the SEM (standard error of the mean) for the data acquired from three independent transfections. Mock samples were prepared by PEG-transfection with no plasmid DNA and (**C**) Flow cytometry histograms (**left** panels) and the images of hemocytometer measurements (**right** panels). The fluorescence intensity on FITC was partitioned into P1 (10^3^–10^4^), P2 (10^4^–10^5^), and P3 (>10^5^) subpopulations, and M means “the missing population”. For a hemocytometer analysis, 10 μL of the transformed protoplasts was mounted on a hemocytometer, and were photographed using the DIC and FITC-A channels of a confocal microscope (200× magnification). The merged images of one square (1 mm^2^) on the hemocytometer were shown. The sGFP-expressing cells were identified as the green fluorescent signals, and the yellow scale bars on the images show the mean intensity ratio (for 10^6^ pixels) of the green fluorescence, analyzed by a Histogram tool.

To determine the effect on a FCA according to each different subcellular localization of sGFPs, the rice protoplasts transfected with each of cytoplasm (sGFP or K-sGFP) or chloroplast (KR-sGFP)-targeting construct were split into four tubes to perform four experiments: (i) a flow cytometric analysis; (ii) quantitative real-time PCR; (iii) western blot analysis; and (iv) hemocytometer measurement. Each analysis was repeated in three independent experiments (Figure 2 and Figure 4).

#### 2.2.1. A Flow Cytometric Analysis (FCA)

In FCA, the fluorescence intensity was normalized by the average value of K-sGFP and showed an eight-fold higher intensity in KR-sGFP as compared to sGFP and K-sGFP (Figure 2A). The transfection ratio was also higher in KR-sGFP (65.7%) than in sGFP (16.8%) and K-sGFP (14.7%), resulting in a 4.5-fold increase in KR-sGFP than sGFP and K-sGFP (see Figure 2B). As shown in Figure 2C, the large difference in the population distribution was observed on the plot data of FCA. The threshold value (10^3^) was determined and the fluorescence below it was regarded as the background signals using mock control. In the case of K-sGFP, a large population (83.3%) was distributed below the threshold value, while the large portion of KR-sGFP (65.7%) was distributed on the overall intervals with higher fluorescence intensity than the threshold value. These results suggest that the localization of sGFP in chloroplasts largely enhances the intensity of fluorescence on FCA (>8-fold) and this causes an increase in the population with higher fluorescence than the threshold level of background signals and, consequently, increases transfection ratio, from 14.7% to 65.7%. In other words, it can be supposed that the population of K-sGFP under the threshold value might contain the transfected protoplasts with lower fluorescence intensity than the background signals. Additionally, the results of FCA between sGFP and K-sGFP are shown to be not considerably different. Thus, it can be supposed that a Kz sequence might not be a much more effective factor for FCA, even if it is helpful to increase the expression level.

**Figure 3:**
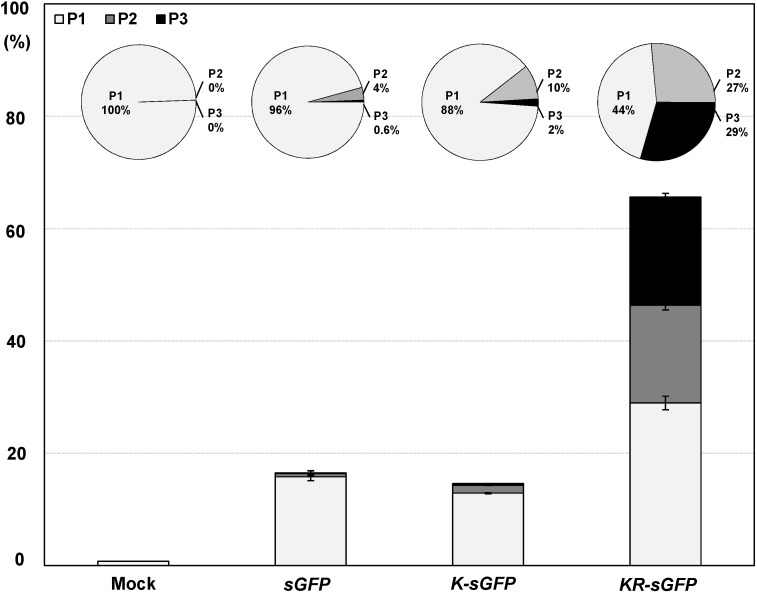
The relative proportional subpopulation distribution of protoplast expressing sGFP. The fluorescence intensity was partitioned into P1, P2, and P3 subpopulations (P1: 10^3^–10^4^; P2: 10^4^–10^5^; P3: >10^5^). The total percentage of protoplasts expressing sGFP on FCA is shown by the sum of percentages of transfected cells on each subpopulation. The circular charts above each bar graph show the ratio of transfected cells on each subpopulation. The transfected protoplast proportion of each subpopulations relative to all transfected protoplasts with a fluorescence intensity exceeding a threshold value (10^3^) was calculated on FCA. The error bars show the SEM (standard error of the mean) for the data acquired from three independent transfections. Mock samples were prepared by PEG-transfection with no plasmid DNA.

To have a deeper look into this difference, the fluorescence intensity was partitioned into P1 (10^3^–10^4^), P2 (10^4^–10^5^), and P3 (>10^5^) subpopulations (see Figure 2C); furthermore, the distribution of transfected cells was compared in those subpopulations. Figure 3 shows that both *sGFP* (96%) and *K-sGFP* (88.4%) of the transfected population (14.4% and 64.3%, respectively) are largely distributed on a P1 subpopulation, while the *KR-sGFP* transfected cells are overall distributed across all subpopulations, P1–P3 and apparently, 55.9% of them belong to P2–P3 subpopulations (>10^4^). It shows that the population with the high fluorescence intensity over 10^4^ was increased by the chloroplast targeting of sGFP. In particular, the population ratio on P3 subpopulation with high fluorescence intensity (>10^5^) was 49- and 14-fold higher in *KR-sGFP* (29.4%) than in *sGFP* (0.6%) and *K-sGFP* (2.1%), respectively.

Taken together, these results suggest that the sGFP targeting into chloroplasts using rRTp plays an important role in improving the fluorescence intensity of FCA to increase the population with higher fluorescence than the threshold value, as well as to increase its average fluorescence intensity (>8-fold). However, the results of hemocytometer analysis (right panels of Figure 2C) do not correspond to the data of fluorescence intensity (left panels of Figure 2C) on FCA, in the point that the histogram analyses of those images showed that the mean intensity ratios between *K-sGFP* and *KR-sGFP* were similarly determined, as shown by scale bars (Figure 2C). Supposedly, the difference of fluorescence intensity on FCA data between *K-sGFP* and *KR-sGFP* might not be derived from the difference on the molecular levels of RNA/proteins or transfection efficiency.

#### 2.2.2. Non-FCA-Based Analyses

**Figure 4:**
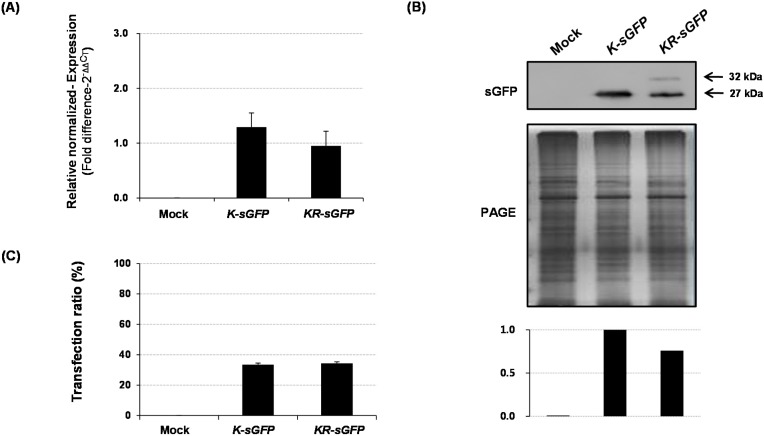
Comparison of *K-sGFP* and *KR-sGFP* constructs based on non-FCA analysis. (**A**) The mRNA transcripts of *sGFP* and *rRTp-sGFP* were compared using real-time PCR. The rice ubiquitin gene was used as an internal reference for quantitative normalization; (**B**) The expression efficiency of proteins compared by western blot analysis. Polyclonal rabbit antibody against sGFP was used at a titer of 1:5000 and the image of PAGE gel was used to show relative quantities of the loaded proteins. The graph shows the relative band intensities quantified using LAS4000 software and (**C**) The ratio of protoplast cells expressing sGFP proteins was analyzed by hemocytometer measurements. A volume of 10 μL of the transformed protoplasts was mounted on a hemocytometer and the images were obtained using the DIC and FITC-A channels of a confocal microscope (200× magnification). The sGFP-expressing cells were identified as the green fluorescent signals. The error bars show the SEM of data acquired from three independent transfections. Control samples were prepared by PEG transfection with no plasmid DNA.

The sGFP expression level and transfection ratio were analyzed using a non-FCA-based analysis (see Figure 4). The sGFP expressions were measured on molecular levels; mRNA transcripts by quantitative real-time PCR (see Figure 4A) and proteins by western blot analysis (see Figure 4B). As a result, both transcripts and proteins of *K-sGFP* FP did not considerably differ from those of the *KR-sGFP*. The *KR-sGFP* formed two protein bands of a premature-polypeptide of 32 kDa and a mature-polypeptide of 27 kDa produced after the cleavage of rRTp-transit peptide. This smaller one from *KR-sGFP* was supposed to be a functional form as a fluorophore emitter (see Figure 4B). The transfection ratio analysis using the hemocytometer showed no difference between *K-sGFP* and *KR-sGFP* (see Figure 4C). These results suggest that the localization of sGFP into chloroplasts does not affect the expression level of sGFP or transfection efficiency and support the presumption that the large population of the *K-sGFP* transfected protoplasts was analyzed as the non-transfected population with background signals because their fluorescence intensity was lower than the threshold value on FCA. The population with the lower fluorescence intensity would henceforth be called “the missing population” (M; see Figure 2C for its interval). This could also be supported by the images of hemocytometer analysis in the right panel of Figure 2C, on which fluorescence intensity and transfection ratio are not much different between *K-sGFP* and *KR-sGFP*.

### 2.3. Optimization of the Amount of Plasmid DNAs

**Figure 5:**
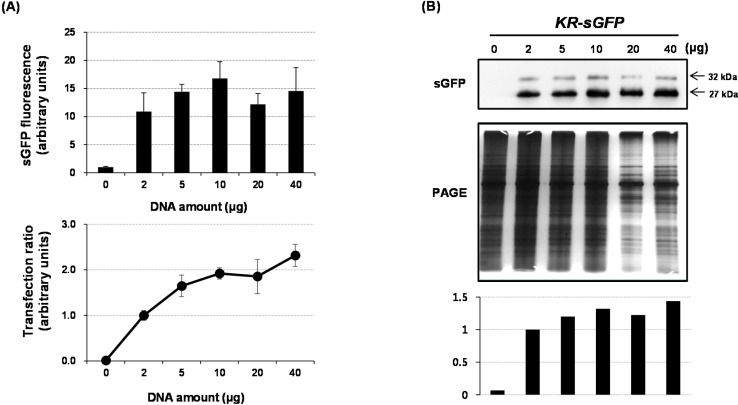
Optimization of the amount of DNA for FCA-based analysis using a *KR-sGFP* reporter system. The fluorescent signals exceeding the gating threshold were detected in single live protoplasts. (**A**) The sGFP fluorescence was detected using the FITC-A channel of the flow cytometer and its values were normalized by the fluorescent intensity of the protoplasts transfected using 0 μg DNA. The results are presented in arbitrary units. The transfection ratio was calculated by the proportion of transfected protoplasts relative to total protoplasts gated by side scattering and forward scattering procedure on FCA. The transfected protoplasts are determined by their fluorescence intensity exceeding the threshold value (10^3^) on FCA; The results are normalized by transfection ratio of the protoplasts transfected using 2 μg DNA and presented in arbitrary units and (**B**) The expression efficiency of proteins was compared using western blot analysis. Polyclonal rabbit antibody against sGFP was used at a titer of 1:5000 and the image of PAGE gel was used to show relative quantities of the loaded proteins. The graph shows the relative band intensities quantified using LAS4000 software.

To optimize the amount of plasmid DNAs for FCA, various quantities (0, 2, 5, 10, 20, and 40 μg) of the *KR-sGFP* plasmid DNAs were used for rice protoplast transformation. The PEG-transfected protoplasts were split into three tubes for three experiments, respectively: (i) FCA (see Figure 5A); and (ii) western blot analysis (see Figure 5B).

FCA showed the lowest sGFP fluorescence at 2 μg DNA and the highest at 10 μg DNA, however, the difference in fluorescence range was not very large among quantities ranging from 5–40 μg (Figure 5A). Similarly, the protein accumulations were not much different among 5–40 μg (Figure 5B). Comprehensively, an optimal amount of transfected DNAs resulted in 10 μg DNAs for FCA. Our results demonstrate that the use of only 5–10 μg DNA was enough for FCA using rice protoplasts and that large amounts of plasmid DNAs (>40 μg) are not necessary for FCA of our protoplast system. In this study, the smallest quantitiy (5 μg DNA) of the optimized DNA quantity (5–10 μg DNA) was used for various analyses.

## 3. Discussion

The powerful capabilities of flow cytometry, including quantification of fluorescence and cell sorting, have been broadly applied in cell biology, molecular biology, and microbiology in various fluid and unicellular systems involving animals and microbes [20,21]. In plant systems, technologies using FCA have also been usefully applied to the comparative promoter analysis and the sorting protoplasts expressing fluorescent proteins [4,5,7]. Recently, technology using FCA has been developed to enable high throughput analysis of more than 10^8^ samples per day [22]. In this study, we developed a useful and powerful strategy of FCA using protoplasts by using efficient translation systems and localization of sGFP into chloroplasts. Chloroplasts have been reported as an excellent reservoir for many kinds of ectopically expressed proteins, in which many kinds of foreign proteins could be kept stable [11]; in rice, 20–30 chloroplasts per a rice mesophyll cell have been reported to exist [23]. Similar numbers of chloroplasts were also shown to be gathered beneath the cell membrane in one protoplast (see Figure 1B).

On FCA, the comparison of fluorescence intensities between *sGFP*/*K-sGFP* (the cytoplasm) and *KR-sGFP* (chloroplasts) showed that chloroplast localization of sGFP increased the average of fluorescence intensity (see Figure 2A). These results could lead to the assumption that the difference in fluorescence intensity might derive from the expression levels. However, the results of real-time PCR and western blot analysis showed incongruously that their expression levels were rather similar to each other (see Figure 4). It suggests that the difference in fluorescence intensity is not related to their molecular levels and might be caused by other factors. Typically, the fluorescence emission (e.g., intensity, spectral features, fluorescence lifetime) are mostly limited to a small number of cellular conditions, such as pH, redox potential or refractive index, temperature, prior illumination, and protein concentration [24] in each protoplast cell, which could affect the emission efficiency of fluorescence from the fluorophore of sGFP. As another important factor that should not be overlooked, we could also consider the self-quenching caused by aggregation of high concentrated fluorophores [25]. However, microscopic analysis using a hemocytometer showed that the fluorescence emission degrees of *K-sGFP* and *KR-sGFP* are not considerably different from each other on the image of microscopic analysis using the same exposure time (see Figure 1B and Figure 2C), suggesting that the difference of fluorescence intensity as much as 8-fold might stem from not only cellular conditions or self-quenching influencing the efficiency of fluorescence emission, but also from other unknown factors.

Previous study has mentioned that the fluorescence intensity of the GFP on non-imaging detection system (e.g., a fluorometry using cuvets or microtiter plates, or a flow cytometry) tends to be inefficiently measured, compared to it on the imaging detection system of GFPs (e.g., a confocal microscopy or a fluorescence microscopy) [24]. This report could partly explain our finding that the fluorescence intensity of *K-sGFP* on FCA was measured much lower than in microscopic imaging analysis. At this point, it is worth noting that an average of 20–30 chloroplasts in rice protoplasts are mostly positioned underneath cellular membrane layers. Cautiously, we could hypothesize that those characteristics of chloroplasts might help to concentrate stable fluorophores and reduce some effects of refractive index and it might consequentially overcome some problems caused by non-imaging detection methods such as FCA. Since the question of how the chloroplast localization of sGFP causes the increase of fluorescence intensity on FCA remains, it should be further analyzed for improving the fluorescence intensity of GFPs on FCA using plant protoplasts.

The transfection ratio of *KR-sGFP* (65.7%) was apparently higher than that of *K-sGFP* (14.7%) (see Figure 2B); however, the hemocytometer measurement showed similar transfection ratio between *K-sGFP* and *KR-sGFP* (see Figure 4C). This suggests that the large portion of protoplasts transfected with *K-sGFP* (cytoplasm) was from data missed on FCA because of their fluorescence intensity lower than the threshold value regarded as the background signal (see Figure 2C). An interval of the missed population was presumably determined between the peak of the *K-sGFP* plot graph and the threshold point (10^3^), called “the missing population (M)” (Figure 2C). In the interval, the height of the plot graph was shown to be largely reduced in *KR-sGFP* compared to *K-sGFP*. These results show that the enhanced intensity of fluorescence (8-fold) by targeting into chloroplasts caused the reduction of the missing population and might be the main reason why the transfection ratios of FCA were different. Moreover, among the *K-sGFP*-transfected protoplasts (14.7%), most (88.4%) were distributed in the first half of the P1 subpopulation (10^3^–10^4^) with the low fluorescence intensity; a mere 2% of them had >10^5^ intensity (see Figure 3). By contrast, the population of *KR-sGFP*-transfected protoplasts (29.4% of total population) with a high fluorescence intensity (>10^5^) was 65.7% of the transfected population (see Figure 3).

Also, since our strategy of FCA using protoplasts is based on targeting fluorescent proteins into chloroplasts, the autofluorescence derived from chlorophylls of chloroplasts should be also considered. It has been reported that plant derived-autofluorescence causes some difficulties with some analytical processes of fluorescence-based techniques, and that chlorophylls of chloroplasts have been considered as one of the strongest autofluorescence contributors [26]. However, several previous reports have shown that only if the commonly used fluorescence markers such as eGFP, YFP and mCherry is used for the fluorescence-based techniques, the autofluorescent background from chlorophylls could be almost spectrally filtered out, because the autofluorescence background of chlorophylls is bathochromically shifted with respect to the fluorescent signals of eGFP, YFP, and mCherry proteins [27,28,29]. Since the fluorescent protein used in this study is a sGFP identical to commercially available eGFP (Clontech; TaKaRa Bio, Shiga, Japan), it could be supposed that the autofluorescence background of chlorophylls is not too much trouble with our strategy of chloroplast targeting-based FCA, because it can be almost filtered out, based on the bathochromic shift among them.

Taken together, our results show that the chloroplast targeting of sGFP caused an increase in the population with high fluorescence intensity (>10^4^) to result in an increase in the average fluorescence intensity; the increase in the population with the fluorescence intensity above the threshold value increased the transfection ratio of FCA. Our strategy using chloroplast targeting is very useful to utilize the advantage of FCA or FACS using protoplasts of green tissues. Also, while a previous study reported DNA-usage in the range of 20–100 μg for transfection [9], our results suggest that only 5–10 μg DNA was enough for FCA. This reduction of DNA-usage on FCA using protoplasts will also be useful for saving time and effort. Likewise, the usefulness of our strategy using rRTp-sGFP should further be investigated with regard to other non-green tissues, such as roots without chloroplasts.

## 4. Materials and Methods

### 4.1. Vector Constructs

The DNA fragments of both rRTp and sGFP were amplified from a pSB-RTG plasmid [14]; the sGFP is identical to commercially available eGFP (Clontech, USA). The Kz sequence (AACAATGGC) identified for plants [16,17,18] and the recombination sites (attB1 and attB2) for a gateway cloning system [30] were introduced as a non-homologous overhang in the designed PCR primers, and the chimeric fragments of *sGFP*, *K-sGFP* and *KR-sGFP* with attB1-B2 sites were prepared by an overlap extension-PCR [31]. The PCR primers used in this study are listed in the Table S1. All PCR reactions were performed using Phusion^®^ High-Fidelity DNA Polymerase (New England Biolabs, Ipswich, MA, USA).

The three chimeric gene fragments (*sGFP*, *K-sGFP*, and *KR-sGFP*) were cloned into the pB2GW7 vector [32] containing a 35S promoter for constitutive expression using a gateway cloning system. Gateway cloning procedures were performed using Gateway^®^ BP Clonase^®^ II Enzyme Mix and Gateway^®^ LR Clonase^®^ II Enzyme Mix following the manufacturer’s instructions (Invitrogen, Waltham, MA, USA).

### 4.2. Plant Materials

The dehulled seeds of rice (*Oryza sativa* L. *Japonica* cv. “Ilmi”) were sterilized by treatment once in 70% ethanol for 1 min and twice in 2% sodium hypochlorite for 20 min and then planted on Murashige and Skoog (MS) agar medium after washing five times with distilled water. The rice seedlings were grown in the dark for 10 days, and then illuminated with white light for 20 h prior to protoplast isolation.

The restricted exposure of the plants to light (20 h) was used to reduce the autofluorescence background resulting from the presence of chlorophyll. The formation of RuBisCo and the biosynthesis of chlorophyll in rice leaves have been reported to be the greatest during greening of etiolated leaf tissues [33,34]. During this process, photosynthesis-related proteins are detected within 2 h of illumination with white light [35], the stromal structures of chloroplast are evident following illumination for 16 h [36], and chlorophyll formation begins subsequently [33,36]. In several preliminary experiments, we established that the optimum illumination time was 16–20 h, which resulted in chlorophyll formation but not to a level where autofluorescence was evident in the images using a fluorescein isothiocyanate (FITC) filter (data not shown). Consequently, 20 h of illumination was used for the FCA analysis of protoplasts in this study.

### 4.3. Protoplast Isolation and Transfection

With minor modifications, protoplasts were isolated as described by Bart [37]. Strips (0.5 mm) were cut with new razor blades from 0.5 g leaf and stem tissue sections and then immediately immersed in 15 mL of enzyme solution (Cellulase R-10 and Macerozyme R-10; Yakult Honsha, Japan) for the digestion of the cell walls. The plate was put into a vacuum desiccator and the vacuum infiltration for 10 min was applied to increase digestion efficiency. Following the incubation with gentle shaking of 50 rpm at RT in the dark for 4 h, the enzyme reaction was stopped by the addition of 15 mL W5 solution [37]. Cell debris were filtered twice through 70 μm and 40 μm BD Falcon™ cell strainers (BD, Franklin Lakes, NJ, USA); furthermore, the protoplasts were pelleted and suspended in Mmg buffer solution [37] at 10^7^ protoplasts·mL^−1^ for PEG-transfection. The number of protoplasts was analyzed using a Marienfeld hemocytometer counting system (Marienfeld-Superior, Berlin, Germany). For transfection, an equal volume of 40% PEG-3350 (Sigma, St. Louis, MO, USA) solution [37] in 0.6 M mannitol and 100 mM CaCl_2_ was added to 110 µL of the protoplasts (around 10^6^ cells) and DNA solution; the mixture was incubated for 15 min. The protoplasts were washed twice with each of the two volumes of W5 and 1 mL of incubation solution and were finally resuspended in 1 mL of incubation solution and incubated at 28 °C in the dark overnight. All plasticware used for protoplasts was coated with 5% calf serum by swirling for 10 s and all buffers were filtered using 0.45 µm syringe filter (Sartorius, Gottingen, Germany).

### 4.4. Microscopy

For the analysis of the subcellular localization, a 5 μg aliquot of each plasmid DNA was transfected into rice protoplasts using PEG-mediated transfection and GFP signals from transfected protoplasts were observed using a Carl Zeiss LSM700 inverted confocal microscope and the image acquisition software ZEN 2009 Light Edition (Carl Zeiss, Oberkochen, Germany). A sGFP fluorescence was detected with 488 nm excitation and 505–530 nm emission wavelengths; the chlorophyll fluorescence was analyzed with 555 nm excitation and >650 nm emission. The fluorescence intensities of images were analyzed using a Histogram tool of the image acquisition software ZEN 2009 Light Edition (Carl Zeiss) following the manufacturer’s instructions. The mean intensities of images were normalized using a *sGFP* construct, and the ratios of the mean intensity were introduced using scale bars in Figure 1B and Figure 2C.

### 4.5. Analysis of sGFP Expression in Protoplasts

For the expression analysis, protoplasts (approximately 1 × 10^6^) were centrifuged, the supernatant was removed, and the pellet was immediately frozen in LN_2_ and stored at −80 °C until RNA and protein extraction.

Total RNA was extracted from rice protoplasts using the RNeasy^®^ Plant mini kit (Qiagen, Hilden, Germany) and the 1st strand cDNA was synthesized using a mRNA selective PCR kit (AMV) ver. 1.1 (Takara, Shiga, Japan) according to the manufacturer’s instructions. Quantitative real-time PCR was performed using a Thunderbird™ SYBR^®^ qPCR mix (Toyobo, Osaka, Japan); the SYBR-fluorescence signals were detected and quantified using a CFX96™ Real-Time PCR system (Bio-Rad, Foster City, CA, USA). The quantified SYBR-fluorescence of *sGFP* transcript was analyzed using CFX Manager™ ver. 2.1 (Bio-Rad). A rice ubiquitin gene (AK061988) was used as a reference gene to normalize the expression data for each *sGFP* transcript.

The protoplast pellet was suspended in 160 μL of phosphate buffered saline (PBS) (Caisson Laboratories, North Logan, UT, USA) and 40 μL of 5× SDS-PAGE loading buffer (Biosesang, Seongnam, Korea) and heated in boiling water for 10 min. The boiled protoplasts were centrifuged at 4 °C; 5 μL of the supernatant was loaded to each of two 12% acrylamide gels for SDS-PAGE. Following the migration, the proteins in one gel were stained with EZ-Silver Staining Kit for Protein (Biosesang) to visualize the loaded amount and quality, while the proteins in the other gel were transferred to PVDF membrane (Whatman, Kent, UK) using Trans-Blot^®^ SD Semi-Dry Electrophoretic Transfer Cell (Bio-Rad). The sGFP chimeric proteins were blotted using rabbit polyclonal antibody of GFP (Abcam, Cambridge, UK) at 1:5000 dilution and secondary anti-rabbit alkaline phosphatase conjugate (Promega, Madison, WI, USA) at 1:5000 dilution. The blots were incubated with Novex^®^ AP Chemiluminescent Substrate (CDP-Star^®^) (Invitrogen). The developed bands were imaged using a LAS-4000 luminescence detector; the intensity of the bands was quantified using the LAS 4000 software (Fujifilm, Tokyo, Japan).

### 4.6. Flow Cytometric Analysis

**Figure 6:**
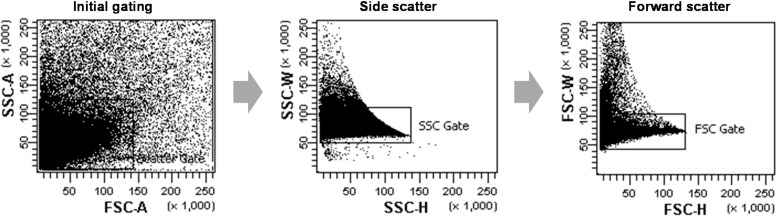
The scatter plots of representative data from FCA of rice protoplasts. The protoplast populations were initially gated through a forward scatter (FSC) area *versus* a side scatter (SSC) area to exclude non-single cells such as cell-multiplets or cell debris. Afterwards, further gates were performed using a SSC-height *vs.* SSC-width dot plot and a FSC-height *vs.* FSC-width to more strictly select single protoplast cells.

The expression of sGFP was monitored at 24 h following transfection using a FACS Aria II flow cytometry system (BD). Protoplasts were pelleted and resuspended in an appropriate volume of PBS buffer and immediately subjected to flow cytometry. Flow cytometry analysis was performed as described by Hagenbeek and Rock [9]. The cell density and sample injection speed was adjusted to the particular experiment for the best possible yield or fastest achievable speed. Live protoplasts were gated using the forward and side scattering fields for data acquisition; irregular shaped protoplasts were regarded as dead or aggregated and were gated out during the data collection. As shown in Figure 6, non-single cells, such as cell-multiplets or cell debris, were excluded through a large initial gating of a side scattering *vs.* a forward scattering (FSC)-A, and two more gating steps of a SSC-height *vs.* -width and a FSC-height *vs.* -width were used more strictly to exclude non-single cells from the next analysis. The green fluorescence of sGFP was excited by a 488 nm laser and detected using the FITC-A channel. For each analysis, the sGFP fluorescence signals of 10^5^ events in the gated population were collected and detected using the FITC-A channel and analyzed using FACS Diva software 6.0 (BD). Control protoplasts were prepared through PEG transfection with no plasmid as a negative control. The fluorescence intensities of the control samples (10^0^–10^3^), derived from chlorophyll in the chloroplasts, provided the autofluorescence background value for FCA of other protoplasts expressing sGFP proteins.

## 5. Conclusions

In this study, the chloroplast targeting transit peptide and Kz sequence for the enhanced translation efficiency were applied to improve the fluorescence intensity of FCA using the rice protoplast system. The localization of sGFP in chloroplasts contributes to the enlargement of the population with the intensity above the threshold value to increase of transfection ratio on FCA from 14.4% to 64.4%. It suggests that the loss of the transfected population is minimized, which is regarded as a non-transfected population, because of their weak fluorescence intensity similar to the background signals. Also, it plays an important role in the increase of population with high intensity (>10^4^) to improve the average of fluorescence intensity. Our strategy could be usefully applicable to various FCAs using green tissue protoplasts.

## Supplementary Materials

Supplementary materials can be found at http://www.mdpi.com/1422-0067/16/01/0788/s1.
